# Prevalence and Predictors of Contraception Usage in Karachi, Pakistan

**DOI:** 10.7759/cureus.11265

**Published:** 2020-10-30

**Authors:** Maheen Siddiqui, Khunsha Fatima, Syeda Nimrah Ali, Mudebbera Fatima, Wajeeha Naveed, Fatima Siddiqui, Tahira Naqvi, Sehar Khan, Mehreen Amin, Amna Liaquat, Zainab Bibi

**Affiliations:** 1 Internal Medicine, Dow University of Health Sciences, Karachi, PAK

**Keywords:** contraception, family planning, condom, pills, iud, contraceptive, unplanned pregnancy

## Abstract

Background

Contraception, also known as family planning, refers to methods that intercept unwanted pregnancies. The modalities of contraception have evolved and multiplied over time to facilitate users. Despite enormous benefits, the contraceptive methods remain underutilized in developing countries such as Pakistan. Accordingly, the primary purpose of our study is to determine the prevalence, perception, and attitude of contraceptive usage in Karachi. The study further assesses the knowledge and core determinants of contraception.

Methods

A cross-sectional study was conducted in Karachi, Pakistan. The sample population comprised currently married males and females residing in Karachi, with females from 16 to 45 years of age and males of 16 years of age and forth. Pregnant females, divorcees, and widowed individuals were excluded. A pre-tested questionnaire was administered to record the demographics, knowledge, and frequency of contraceptive usage, and attitude and perception towards contraception. The data were analyzed using the Statistical Package for the Social Sciences (SPSS( v. 23.0 (IBM Corp. Armonk, NY).

Results

Results depicted that 93.4% (n=468) had knowledge of contraception while 49.7% (n=249) were currently using contraception. The most frequently employed contraceptive modality emerged as condoms (n=163, 65.5%), followed by withdrawal (n=71, 28.5%) and pills (n=62, 24.9%). The prevalence of contraceptive usage was more inclined towards the educated stratum and higher socioeconomic class. Potential predictors for contraceptive usage came out as the number of children, knowledge regarding contraception, spouse’s support for usage, a desire for more children, and family planning counseling (p<0.05).

Conclusion

There is a drastic increase in the prevalence of contraceptive usage in Pakistan as compared to previous studies. Efforts must be made to further augment the knowledge and awareness regarding contraceptive modalities to increase acceptability and eliminate myths prevalent in society regarding contraception.

## Introduction

Contraception methods are key to preventing unwanted pregnancies and sexually transmitted diseases (STDs). Contraception, or family planning, promotes gender equality by providing women with opportunities to free themselves from the unwanted burden of menstrual cycles, breastfeeding, and childcare. Increased parity results in an increase in the number of unsafe abortions and maternal mortality due to multiple risk factors such as elderly age, reduced birth spacing, increased prematurity, and other obstetric emergencies. In 2012, Lancet published that increase in contraception usage in developing countries has shown a decrease in maternal mortality by 40% over the past 20 years, by reducing unintended pregnancies and preventing high-risk pregnancies [1]. Nevertheless, according to the World Health Organization (WHO) 2018 family planning factsheet, 214 million women of reproductive age in developing countries are not using a modern contraceptive method despite their desire to avoid pregnancy [2].

According to the Pakistan Demographic and Health Survey (PDHS) 2012-2013, Pakistan is the sixth most populous country in the world [3]. Not only is maternal mortality high in Pakistan but also the neonatal mortality rate (NMR) has remained virtually unchanged as highlighted by the Pakistan Demographic and Health Survey (PDHS) 2006-2007 [4]. The national abortion rate was 50 per 1,000 women aged 15-49 years in 2012, which was considerably higher as compared to the 2002 statistics [5]. Twenty percent of married women of reproductive age in Pakistan have an unmet need for contraception while the country's contraceptive prevalence rate (CPR) is only at 30%. It has also been documented that more than one-third of women who started using a contraceptive method in the last five years had discontinued it within 12 months. The usage of family planning in rural areas of Pakistan is only 31%, with urban areas showing a slight increase in this regard (around 45%). Collectively, the usage of modern and effective family planning methods in Pakistan account for only 26% of the population. Among modern family planning methods, tubal ligation is the most common method typically acquired in late age, after about 31.5 years [3].

A thorough literature survey was done using the keywords “contraception prevalence and predictors” in the PubMed database and Google Scholar. The literature search revealed ample studies worldwide, with considerable disparities in terms of predictors of contraceptive usage due to inter-individual, cultural, and demographic differences. However, in Pakistan, although the low prevalence of contraceptives has been well-documented, studies observing public perception regarding contraception are still lacking. Few studies have assessed contraception trends in a few cities and local regions of Pakistan, where access to family planning services, family size, employment and educational status, cultural and religious beliefs, sex preferences, reproductive health concerns, and perception about side effects emerged as significant predictors of major acceptance or avoidance of contraception. Considering a finding from a study conducted in India, there existed a major contrast in contraceptive prevalence and perception among different regions of the same country. Under the premise, our literature search did not reveal sufficient data regarding public perception of contraceptive usage across various cities of the country. Moreover, very few studies have considered both males and females alike for determining the contraception trends.

Accordingly, the primary objectives of this study were to determine the prevalence of contraception usage and to assess the general perception and attitude of the public regarding contraception in Karachi, Pakistan. Our secondary objectives were to assess the knowledge and awareness of people about various contraceptive methods and to investigate the key determinants and factors leading to both usage and avoidance of contraception. We further aimed to determine the proportion of individuals who refer to healthcare settings for family planning decisions.

## Materials and methods

Study type and location

A descriptive, cross-sectional study was conducted to assess the prevalence and predictors of contraception usage in Karachi, Pakistan. For this purpose, residents of the city were selected using a non-probability convenience sampling technique. The study was conducted through January 2020.

Sample size and study population

The sample size was calculated using OpenEpi (www.OpenEpi.com), with an anticipated frequency of contraception use of 35.4% with a 95% confidence interval and a margin of error of 2.5% [3]. The computed sample size was 352; however, a total of 550 individuals were surveyed across different localities of the metropolis. In total, 49 participants failed to complete the survey, giving a response rate of 91.1%.

Eligibility criteria

Study participants included currently married males and females residing in Karachi, with females between 16 and 45 years of age and males of at least 16 years of age. Exclusion criteria included pregnant females, divorcees, widowed individuals, males with wives over 45 years of age reaching or having reached menopause, and spouses of participants having already filled the questionnaire. Individuals not permanently residing in the city were also excluded from the study.

Study tool

The study was conducted using a 19-item questionnaire. The questionnaire was designed after a meticulous literature review on the subject matter, having adopted components from similar studies conducted in Bangladesh and Pakistan [6-7]. The questionnaire was reviewed by two medical professionals and was subjected to pilot testing to evaluate its validity and comprehensibility. The survey was then distributed amongst 20 individuals based on convenience of allocation and was modified and remodeled per the results. It was further translated to Urdu, the native language, to facilitate participants.

The survey comprised three sections. The first section documented the basic demographics of the respondents, including self and spouse’s age, education, occupation, age at marriage, number of children, and monthly household income. Socioeconomic status was estimated using quintiles based on monthly household income from the Household Integrated Economic Survey conducted by the Pakistan Bureau of Statistics for the year 2015-2016, with the first quintile representing the lowest-earning population and the fifth quintile representing the highest earners [8]. Ethnic heterogeneity was also taken into account under the premise that it served as a potential predictor. The second section evaluated the knowledge and usage of contraception amongst the respondents. Questions were administered under the section to establish whether or not, and the extent to which, the respondent was aware of contraception; the mode of acquisition of knowledge regarding contraceptive modalities, and the frequency of usage were also assessed. The third section appraised the perception and attitude of respondents toward contraception. Reason(s) for contraception usage/non-usage, associated myths, potential side effects, and positive and negative attitudes toward contraception were explored.

Informed written consent was obtained prior to participation in the study. The participants were then asked to fill out the questionnaire. Participants who could not read or write were interviewed face-to-face following a standard operating protocol employed by all interviewers to avoid plausible bias. Incomplete responses were discarded. No imputation methods were used.

Data analysis

Data entry and analysis was done using the Statistical Package for Social Sciences 23.0 (IBM Corp., Armonk, NY). A knowledge score was worked out to gauge the respondent’s understanding regarding different contraceptive modalities. A total score of 12 was established; a score below six connoted “poor” knowledge, a score of seven to nine indicated “average” knowledge, and a score of 10 and above conferred a “good” knowledge score. Categorical variables were expressed using frequencies and percentages. The Pearson chi-square test was used to compare categorical variables. Statistical significance was set at p<0.05.

## Results

Sociodemographic characteristics

A total of 501 participants responded to the questionnaire; three-fifths of whom were females (n=305, 60.9%). Of the respondents, approximately half belonged to the age group of 26-35 years (n=265, 52.9%) with a median age of 30 years. The majority of the participant’s age at marriage was 18-25 years (n=276, 55.1%) with a median age of 23 years. A total of 183 respondents had completed university education (36.5%) while 117 of the spouses of participants had completed university education (34.7%). Most of the participants followed Islam (n=467, 93.2%). One-fifth of the subjects belonged to Sindhi ethnicity (n=102, 20.4%), and 219 of the respondents had more than two children (43.7%). About 39.9% of the respondents had a total income between Rs.0 and Rs. 22,500 (n=200) (Table 1).

**Table 1:** Summary of sociodemographic characteristics

Variables	Frequencies (%)
Gender	
Male	196 (39.1)
Female	305 (60.9)
Education of Participant	
University	183 (36.5)
Higher Secondary School	62 (12.4)
Secondary School	74 (14.8)
Primary School	69 (13.8)
No Formal Education	113 (22.6)
Age of Participant	
10-17 years	2 (0.4)
18-25 years	93 (18.6)
26-35 years	265 (52.9)
36-45 years	121 (24.2)
45-70 years	20 (4)
Age of Participant at Marriage	
10-17 years	58 (11.6)
18-25 years	276 (55.1)
26-35 years	160 (31.9)
36-45 years	5 (1.0)
45-70 years	1 (0.2)
Religion	
Islam	467 (93.2)
Christianity	13 (2.6)
Hinduism	21 (4.2)
Other	0 (0.0)
Ethnicity	
Sindhi	102 (20.4)
Punjabi	64 (12.8)
Pathan	59 (11.8)
Baloch	33 (6.6)
Mahajir	157 (31.3)
Other	86 (17.2)
Number of Children Alive	
<2 children	212 (42.3)
>2 children	219 (43.7)
Total Income (All Sources Combined)	
Rs. 0-22,500	200 (39.9)
Rs. 22,500-27,000	22 (4.4)
Rs. 27,000-32,000	39 (7.8)
Rs. 32,000-50,500	50 (10.0)
Rs. 50,500-700000	189 (37.7)

Factors associated with current contraceptive usage

A significant correlation was found between current contraception usage and level of education of the participant and spouse (p<0.05), monthly household income (p=0.001), number of children (p=0.024), and knowledge of contraception (p<0.001). The research also yielded a significant relationship between contraceptive usage and spouse’s support for contraception (p<0.001), a desire for more children (p<0.001), family planning counseling (p=0.024), and consensus family planning decisions (p<0.001). No significant relationship was derived between current contraception usage and gender, age, religion, the ethnicity of the participant, and the number of prior unplanned pregnancies (p>0.05).

Knowledge, practice, and factors associated with contraceptive modalities

Of the respondents, 468 had knowledge of contraception (93.4%) while 179 had used contraception in the past (35.7%). Two-hundred forty-nine (249) of the respondents were currently using contraception (49.7%). Furthermore, educated respondents (university qualification) were more prone to using contraception than uneducated ones (no formal education). Contraceptive usage was more prevalent in the higher socioeconomic stratum (n=189, 37.7%) while the methods of contraception most known to the respondents were condoms (n=432, 92.3%), followed by pills (n=399, 85.3%) and withdrawal (n=305, 65.2%). Half of the respondents had poor knowledge (n=247, 49.3%), one-fourth had good knowledge (n=136, 27.1%), and the rest (n=118, 23.6%) had average knowledge of contraceptive modalities.

Through this study, it was observed that there is a significant correlation between knowledge of contraception and discussion of family planning with health care professionals (p<0.001). Moreover, the basic mode of acquisition of knowledge regarding contraceptive modalities in two-fifths of the respondents was through family, friends, and relatives (n=278, 55.5 %).

The most common form of contraceptives used were condoms (n=163, 65.5%), withdrawal (n=71, 28.5%), and pills (n=62, 24.9%) (Table 2).

**Table 2:** Knowledge and practice of various contraceptive modalities IUD: intrauterine device

Variables	Practice of type of contraception	Knowledge of type of contraception
	Frequencies (%)	Frequencies (%)
Condoms	163 (65.5)	432 (92.3)
Pills	62 (24.9)	399 (85.3)
IUD	30 (12.0)	303 (64.7)
Withdrawal	71 (28.5)	305 (65.2)
Periodic Abstinence	27 (10.8)	239 (51.1)
Injectables	18 (7.2)	299 (63.9)
Implants	7 (2.8)	225 (48.1)
Lactational Amenorrhea	9 (3.6)	253 (54.1)
Standard Day Method	11 (4.4)	190 (40.6)
Emergency Contraception	6 (2.4)	201 (42.9)
Female Sterilization	20 (8.0)	266 (56.8)
Male Sterilization	1 (0.4)	204 (43.6)

The most common reason for contraceptive usage was to prevent unwanted pregnancy (n=141, 28.1%), followed shortly after by child spacing (n=134, 26.7%), and having achieved the desired number of children (n=85, 17%). Other reasons included ongoing breastfeeding, recent marriage, financial instability, work orientation, and having prior children with congenital diseases. Among the reasons for non-usage, the reasons encountered were desire for more children (n=93, 18.6%), fearing the side effects of usage (n=50, 10%), difficulty getting pregnant (n=23, 4.6%), and religious prohibition (n=21, 4.2%) (Table 3).

**Table 3:** Assessing the reasons for contraceptive usage and non-usage STD: sexually transmitted disease

Variables	Frequencies (%)
Reasons for Contraception Usage	
Prevention of unwanted pregnancy	141 (28.1%)
STD prevention	3 (0.6%)
Child spacing	134 (26.7%)
Desired number of children achieved	85 (17%)
Reasons for Contraceptive Non-Usage	
Desire for more children	93 (18.6%)
No/infrequent sex	15 (3%)
Breastfeeding	8 (1.6%)
Difficulty getting pregnant	23 (4.6%)
Post-partum state	3 (0.6%)
Religious prohibition	21 (4.2%)
Reduced sexual pleasure	7 (1.4%)
Fear side effects	50 (10%)

Attitude - myths, side effects, and beliefs associated with contraception

Fifty-five point nine percent 55.9% (n=280) of the respondents believed that there were no side effects of contraception usage. The most common side effects/myths that people reported were health problems (n=254, 50.7%), weight gain (n=241, 48.1%), displeasure of God (n=200, 39.9%), and cultural disapproval (n=174, 34.7%).

Three-hundred thirteen (313) of the respondents (62.5%) found contraception to be beneficial, 278 found it safe (55.5%), 238 (47.5%) reliable, and 153 (30.5%) pleasant (Table 4).

**Table 4:** Myths, side effects, and beliefs associated with contraception

Variables	Yes	No	Do not know
	Frequencies (%)	Frequencies (%)	Frequencies (%)
Weight gain	241 (48.1)	112 (22.4)	148 (29.5)
Birth defects	126 (25.1)	202 (40.3)	173 (34.5)
Infertility	149 (29.7)	198 (39.5)	154 (30.7)
Abortion	150 (29.9)	194 (38.7)	157 (31.3)
Reduced sexual pleasure	124 (24.8)	207 (41.3)	170 (33.9)
Cancer	97 (19.4)	205 (40.9)	199 (39.7)
Cultural disapproval	174 (34.7)	230 (45.9)	97 (19.4)
Displeasure of God	200 (39.9)	205 (40.9)	96 (19.2)
Health problems	254 (50.7)	139 (27.7)	108 (21.6)
Beneficial	313 (62.5)	75 (15)	113 (22.6)
Safe	278 (55.5)	96 (19.6)	127 (25.3)
Reliable	238 (47.5)	111 (22.2)	152 (30.3)
Pleasant	153 (30.5)	169 (33.7)	179 (35.7)

One-hundred eighty-two (182; 36.3%) of the respondents had a history of an unplanned pregnancy. Two-hundred ninety-eight (298; 59.4%) desired more children, with 17.5% (n=52) respondents desiring a male child, 9% (n=27) desiring a daughter, and the majority (n=219, 73.5%) being fine with either. Most of the respondents (n=401, 80.0%) made mutual decisions regarding family planning. Three-fifths of respondents (n=299, 59.7) did not consult a health care professional regarding family planning, with a mere 7% (n=35) having no access to a healthcare professional (Table 5).

**Table 5:** Family planning decisions and counseling

Variables	Frequencies (%)
History of unplanned pregnancy	182 (36.3)
Desire for more children	298 (59.4)
Male	52 (17.5)
Female	27 (9)
Both	219 (73.5)
Family Planning Decision Lies On	
Participant	57 (11.4)
Spouse	43 (8.6)
Both	401 (80.0)
Family Planning Counseling by Health Care Professional	
Yes	167 (33.3)
No	299 (59.7)
No access to health care professional	35 (7.0)

## Discussion

Contraception is the central pillar of family planning. Modern contraceptive methods and family planning have played a pivotal role in the economic development and progress of various developed nations across the globe. Nonetheless, despite the advent of the modern methods of contraception and their easy availability, population growth has not decreased proportionally in the developing world.

In our study population of 501 participants, with 60.9% (n=305) being females and 39.1% (n=196) being males, only 35.7% (n=179) of the participants had used contraception in the past, with only 49.7% (n=249) currently using contraceptive methods. Thus, nearly half of our study population employed contraceptive modalities with the other half using no modality at all. As compared to a study conducted in Pakistan in 2013, this reflects a drastic increase in the prevalence of contraception usage in the region, with only 17% of the population using contraception in the past [9].

Regarding knowledge of contraceptive modalities, 49.3% (n=247) of the participants had poor knowledge regarding modern methods. Of our study population, 36.5% (n=183) of the participants were university graduates and 34.7% (n=117) of the participants’ spouses were of university-graduate educational level, rendering a precise pattern of approach and significant association with contraceptive usage. The educated participants in our study were more prone to using modern contraceptive methods than their uneducated counterparts. This corroborates with previous studies that also established a direct relationship between the level of literacy of the population and the usage of contraceptive methods [6]. A plausible explanation for the trend observed could be that the uneducated proportion of our population is less prone to have access to knowledge regarding different methods of contraception and to the health and economic advantages of birth spacing [10-11].

Our results also discovered that the probability of contraceptive usage was greatest among participants who had availed of family planning services. A significant number of participants (n=299, 59.7%) claimed never having consulted a healthcare professional for family planning, whereas only 7% (n=35) of participants stayed devoid of healthcare setups. This, again, can be attributed to a lack of knowledge and awareness regarding contraceptive methods and to the innumerable misconceptions and myths held by our population against contraceptive usage. Consistent with our results, a previous study conducted in Pakistan also found that women who accepted family planning services or those who acknowledged the public advertisements of contraceptive methods were more likely users of modern contraceptive methods, unlike those who did not consult family planning services at all [12].

The most common method of contraceptive modalities used were condoms (n=163, 65.5%), followed by withdrawal (n=71, 28.5%) and pills (n=62, 24.9%). Previously, studies by Qazi et al. and Donadiki et al. also reported condoms as the most prevalent method of contraception used among their respective study populations [13-14]. A possible explanation for this could be the greater knowledge of this method in the population through TV advertisements, friends, and families. Also, condoms are easy to use and widely available at a low cost, prompting their greater use and subsequent compliance when compared to other methods.

Our study also established that a staggering 0.6% (n=3) of our population had knowledge regarding protection against sexually transmitted diseases rendered by using barrier contraceptive methods while the rest of the population was entirely oblivious to the fact.

We observed that the most common reason for using contraception was the prevention of unwanted pregnancy (n=141, 28.1%). This explains that the primary reason for the couples to use contraception is to acquire the desired family size. A previous study has shown that the number of alive children greatly influences the decision of using contraception; couples who have completed their families and have healthy children are more likely the users of different methods of contraception as compared to newly married couples or those with a history of abortions, child deaths, and children with diseases [15]. On the contrary, the most common reason for the non-usage of contraception was a desire for more children (n=93, 18.6%). Other reasons observed for not using contraception were mainly misconceptions and myths.

The most common side effects dreaded were health problems (n=254, 50.7), followed by weight gain (n=241, 48.1%). Another reported misconception in our study was the fear of getting infertile after ceasing the use of the contraception method (n=149, 29.7%). This aligns with a study by Ngum et al. who researched the knowledge and attitudes regarding contraception among African American teenage mothers and reported a fear of getting infertile as the most common misconception in the study population [16]. Other misconceptions encountered in our study included the presumed side effects of contraception like abortion (n=150, 29.9%), birth defects (n=126, 25.1%), reduced sexual pleasure (n=124, 24.8%), and cancer (n=97, 19.4%).

A significant proportion of the population avoided the use of contraception because of myths prevailing in society passed down from one generation to another. The most common myths dealt with in our study were displeasure of God (n=200, 39.9%) and cultural disapproval (n=174, 34.7%). This signifies that there is a substantial impact of religious, cultural, and social beliefs on the use of contraception in the population. Many participants who wanted to avoid unwanted pregnancies and had larger families than planned did not use contraception because of religious and cultural disapproval. In our population, 93.2% (n=467) were Muslims, 4.2% (n=21) were Hindus, and 2.6% (n=13) were Christians. Interestingly, a prior comparative analysis showed that Muslim women and followers of other traditional religions were least likely the users of contraception [17]. As our population was Muslim predominant, it is probable that myths regarding modern contraceptive methods are more prevalent in this population. 

We conclude that the level of literacy, the acceptability of family planning services, religious beliefs, and social and cultural attitudes greatly influence the preferences of contraceptive methods and decision on contraception usage. There must be efforts made to improve the knowledge and awareness of contraception among the people of Pakistan. Better education and awareness would improve the approach towards contraception and would contribute toward population control and thus economic development. We also recommend that more studies at larger scales should be conducted for a better understanding of the knowledge and attitudes of the Pakistani population regarding contraception.

Our study is subject to certain limitations. Firstly, only married couples were included in our study, thus we could not study the contraception knowledge and practices of unmarried sexually active individuals. Secondly, multiple interviewers collected data, thus interviewer bias cannot be ruled out.

## Conclusions

The study reflects good knowledge of the population regarding contraception. Interestingly, however, the prevalence of contraception usage remains limited to half the population. Efforts should, therefore, be made to augment the understanding of contraception to greater depths, thus enhancing the level of acceptability of the public toward contraception. Mass media and the healthcare sector should deploy joint efforts in this regard to promote acceptability, addressing the age-long myths and misconceptions to bring about a definite change.
